# Current epidemiology of diabetic retinopathy in patients with type 1 diabetes: a national multicenter study in Brazil

**DOI:** 10.1186/s12889-018-5859-x

**Published:** 2018-08-08

**Authors:** Laura Gomes Nunes Melo, Paulo Henrique Morales, Karla Rezende Guerra Drummond, Deborah Conte Santos, Marcela Haas Pizarro, Bianca Senger Vasconcelos Barros, Tessa Cerqueria Lemos Mattos, André Araújo Pinheiro, Felipe Mallmann, Franz Schubert Lopes Leal, Fernando Korn Malerbi, Marilia Brito Gomes

**Affiliations:** 1 Department of Ophthalmology, State University of Rio de Janeiro, Avenue Boulevard 28 de Setembro, 77, 4th floor, Rio de Janeiro, CEP 20.551-030 Brazil; 2 Department of Ophthalmology, Federal University of São Paulo, São Paulo, Brazil; 3 Department of Ophthalmology, Hospital Federal dos Servidores do Estado, Rio de Janeiro, Brazil; 4 Department of Internal Medicine, Diabetes Unit, State University of Rio de Janeiro, Rio de Janeiro, Brazil; 5 Department of Ophthalmology, Centro de Endocrinologia e Diabetes do Estado da Bahia, Salvador, Brazil; 6 Department of Ophthalmology, Hospital Regional de Taguatinga, Brasília, Brazil; 7 Department of Ophthalmology, Federal University of Rio Grande do Sul, Porto Alegre, Brazil; 8 Department of Ophthalmology, University of Campinas, Campinas, Brazil; 9 Department of Endocrinology and Ophthalmology, Federal University of São Paulo, São Paulo, Brazil

**Keywords:** Retinopathy, Epidemiology, Microvascular disease, Type1 diabetes, Vision-threatening, Risk factors, Uric acid

## Abstract

**Background:**

Diabetic retinopathy is the leading cause of blindness in economically active populations. The aims of this study were to estimate the prevalence and to identify risk factors for diabetic retinopathy in patients with type 1 diabetes in Brazil.

**Methods:**

This was a nationwide, cross-sectional study conducted between August 2010 and August 2014. The study included 1760 patients with type 1 diabetes. Patients underwent a standard questionnaire, clinical and laboratory analyses and were screened for diabetic retinopathy. To analyze the risk factors related to diabetic retinopathy, two models of logistic regression models were performed, one considering vision-threatening cases and the other with any diabetic retinopathy cases as dependent variables. The group with vision-threatening included patients with severe non-proliferative diabetic retinopathy, proliferative diabetic retinopathy and macular edema.

**Results:**

In total, 1644 patients (mean age, 30.1± 12.0 years; duration of diabetes, 15.3 ± 9.3 years; female, 55.8%) were studied. 35.7% presented diabetic retinopathy and 12% presented vision-threatening diabetic retinopathy. Three risk factors associated with diabetic retinopathy were in common to both groups: longer diabetes duration (OR 1.07; 95% CI, 1.05–1.09), higher levels of HbA1c (OR 1.24; CI, 1.17–1.32) and higher levels of serum uric acid (OR 1.22; CI, 1.13–1.31) (*p* < 0.001 for all comparisons).

**Conclusion:**

The higher rate of vision-threatening retinopathy found in our study highlights the need to improve access to eye care and screening programs for diabetic retinopathy in Brazil. In addition to traditional risk factors, we found an association between serum uric acid levels and diabetic retinopathy. Further studies are needed to address this association.

**Electronic supplementary material:**

The online version of this article (10.1186/s12889-018-5859-x) contains supplementary material, which is available to authorized users.

## Background

Type 1 diabetes mellitus (T1D) is a chronic metabolic disease that is characterized by sustained hyperglycemia that leads to macro and microvascular complications, such as retinopathy, nephropathy and neuropathy [1]. Diabetic retinopathy (DR) is considered the leading cause of blindness in economically active populations worldwide and represents a significant social and financial burden for the patients and healthcare systems [2]. The combination of an increasing incidence of T1D worldwide [1, 3] and the aging of populations is resulting in an increase in diabetes-related complications, including DR. The worldwide prevalence of DR has been estimated to be 35%, and the prevalence of vision-threatening DR (VTDR) has been estimated to be 10% [4, 5]. VTDR may soon result in vision loss if left untreated.

DR represents a retinal pathology that may closely reflect microvascular damage in other vascular beds and is considered an important indicator of microvascular complications [6]. Furthermore, fundus examination is an inexpensive and non-invasive opportunity to access the microvascular bed.

The duration of diabetes and poor glycemic control are the most important risk factors associated with the development of DR and the majority of diabetes-related chronic complications. Important clinical trials, such as *The Diabetes Control and Complications Trial*/*Epidemiology of Diabetes Intervention and Complications* (DCCT/EDIC) have demonstrated that intensive treatment of hyperglycemia effectively delays the onset and slows the progression of complications in T1D, including DR [7]. However, other risk factors have been associated with DR, including nephropathy, hypertension and dyslipidemia [2].

Screening for DR is an important tool for detecting VTDR and preventing blindness. Currently, approximately only 60% of patients with diabetes have yearly screenings for DR [5]. However, recent studies have shown a tendency towards individualized retinal screening intervals based on glycated hemoglobin levels, type and the duration of diabetes [8].

Systematic screening for DR is important to identify patients who need referral to a specialist, especially those with VTDR thereby preventing blindness and visual impairment [9–11]. However, Brazil, along with several other developing countries, does not have a national screening program for DR, neither, as far as we know, data on its prevalence.

The purpose of this study was to estimate the prevalence of and the risk factors for DR based on a nationwide study of T1D in Brazil.

## Methods

### Study design

This report describes a multicenter, nationwide, cross-sectional study including 1760 patients that was conducted between August 2010 and August 2014 in 14 secondary public centers (ambulatory outpatient clinics) and tertiary care centers (ambulatory outpatient clinics in university hospitals) located in the urban areas of 10 cities, representing all Brazilian geographic regions. The detailed methodology has been described previously [12]. Briefly, all patients received public healthcare from the National Brazilian Health Care System (NBHCS), and each clinic reported data for at least 50 consecutive type 1 diabetes patients who regularly attended the clinic.

The diagnosis of T1D was done by an endocrinologist in the presence of characteristics signs of TD1, such as polydipsia, weight loss, polyuria, variable degrees of hyperglycemia and the need for continuous insulin use since the diagnosis. Inclusion criteria were patients older than 13 years, followed at each center for more than 6 months. Patients who had had diabetic ketoacidosis or infectious diseases in the last 3 months, as well as pregnant and lactating women, were excluded. The ethics committees of each participating center, in addition to the ethics committee of the coordinating center, at the Pedro Ernesto University Hospital, at the State University of Rio de Janeiro, approved the study. The informed consent term was signed by patients or their legal guardians. The research was conducted in accordance with the Helsinki Declaration.

The quality of the study was assessed using the checklist “Strengthening the Observational Report on Epidemiology” (STROBE) [13].

### Data collection

#### Clinical data

The patients were submitted to a questionnaire standardized by a trained physician. Patients characteristics included height (m), age at diagnosis, age, diabetes duration, weight (kg), abdominal circumference, adherence to diet, insulin therapeutic regimen, smoking status, personal and family medical histories, economic status and number of years of formal education.

The definition of smoking was based on the current use of more than one cigarette per day.

Hypertension in adults was self-reported. We consider hypertensive patients who report a previous diagnosis of hypertension on at least two different occasions, by a health professional.

For the definition of obesity, we considered the body mass index (BMI) ≥ 30 kg / m^2^ and overweight a BMI ≥ 25 kg / m^2^.

The Macrovascular disease was defined by the presence in the patient medical record of one or more of the following conditions: coronary artery bypass surgery, angina, coronary angioplasty, myocardial infarction, peripheral vascular disease or stroke.

#### Laboratorial data

To analyze the blood samples, high-performance liquid chromatography (HPLC, Bio-Rad Laboratories, Hercules, California, USA) was used for glycemia and for glycated hemoglobin (HbA1c), enzymatic techniques was used for cholesterol (total, HDL and LDL and triglycerides). We adopted the following ADA parameters for adequate clinical and metabolic control [2, 14]: good glycemic control was defined as HbA1c < 7.0% (53 mmol/mol) for adult and < 7.5% (58 mmol/mol) for adolescents and poor glycemic control was defined as HbA1c ≥ 9.0% (75 mmol/mol), for a good control of cholesterol were considered the values of total cholesterol < 200 mg/dl (5.2 mmol/L), LDL cholesterol < 100 mg/dL (2.6 mmol/L), HDL cholesterol > 50 mg/dL (1.3 mmol/L) for women and > 40 mg/dL for men (1, 1 mmol/L), and triglycerides < 150 mg/dL (1.7 mmol/L).

A commercial urease-based kit (BioSystem) was used to measure serum uric acid (SUA) and the following normal values were considered: 2.6–6.0 mg/dl in women and 3.5–7.2 mg/dl in men.

Albuminuria was measured in two morning urine samples with a minimum interval of 1 week and a maximum of 6 months. The values of the urinary albumin were expressed in means (mg/dl) and the dosage was performed by immunoturbidimetry. We used the CKD-EPI equation [15] to estimate renal function and expressed it as a glomerular filtration rate (GFR) in units per milliliters per minute per 1.73 m^2^ (ml/min). We defined chronic kidney disease (CKD) when albuminuria ≥30 mg/dl and GFR < 60 ml/min [2].

#### Diabetic retinopathy data

DR screening was accessed by mydriatic binocular indirect ophthalmoscopy (BIO), which was performed in each center, by an ophthalmologist specialized in retina, who was trained before the beginning of the study in an ophthalmologic university center. The stage of retinopathy for each patient was considered by the eye with more severe retinopathy. Each eye was classified based on the absence or presence of DR. Patients with DR are classified considering the international classification of DR as: non-proliferative diabetic retinopathy (NPDR) mild, moderate or severe; proliferative diabetic retinopathy (PDR) and macular edema [16]. Additionally, DR was categorized as VTDR (severe NPDR, PDR, and macular edema) or non-VTDR (absent of DR, mild NPDR and moderate NPDR, without macular edema).

#### Sample data calculation and economic status

In order to determine the sample size of this study, the distribution of the Brazilian population in the different geographic regions was taken into account, according to the population distribution reported by the 2000 Brazilian Institute of Geography and Statistics Census (IBGE) [17]. The calculation of this sample is already described previously in the Brazilian Multicenter Type 1 Diabetes Study [12].

Centers included in our study were all from urban areas because the vast majority of type 1 diabetes patients are treated on reference centers located in urban areas. It is important to note that, based on the IBGE census, the majority of the Brazilian population (approximately 85%) resides in urban areas.

The economic status was stratified in the categories of high, medium, low and very low income, according to the Brazilian Economic Classification Criteria [18].

### Statistical analysis

We conducted descriptive analyzes to evaluate the associations between demographic, clinical and laboratory data with DR. The Student t test or the ANOVA with Sidak correction were used for continuous variables. The chi-square test was used for the categorical variables. We present the data as frequencies (percentages) for the categorical variables and as the standard deviation of the means (SD) for the continuous variables.

Univariate analysis was performed to evaluate the relationship between RD and its risk factors. We included in the multivariate analysis the variables with *p* < 0.1 in the univariate and some variables of interest like gender and economic status. Two models of logistic regression were performed: one model for each considered category of DR as the dependent variable. First, we considered any DR vs. the absence of DR; secondly, we considered VTDR vs. non-VTDR (absent, mild NPDR and moderate NPDR). For each model, we tested the same covariates, including gender, age, duration of diabetes, economic status, years of formal education, HbA1C, CKD (yes/no), hypertension (yes/no), use of angiotensin-converting enzyme (ACE) inhibitor (yes/no), serum uric acid (SUA), HDL cholesterol, LDL cholesterol, triglycerides, BMI, macrovascular disease (yes/no), and smoking status (yes/no). The exploratory Forward-Wald stepwise regression was performed, and it was determined which variables contributed more to the discrimination between groups. The Nagelkerke R-squared value was also calculated.

All analyses were performed using the Statistical Package for the Social Sciences (SPSS version 20, SPSS, Inc., Chicago, IL, USA). Odds ratios (ORs) with 95% confidence intervals (CIs) were calculated when indicated. A two-sided *p* < 0.05 was considered statistically significant.

## Results

### Prevalence of diabetic retinopathy

Of the 1760 patients recruited at baseline, a total of 1644 (93.4%) were screened for DR with fundoscopy and were included in this study. Of these patients, 1055 (64.2%) had no retinopathy and 589 (35.7%) presented with DR as follows: 298 (18.1%) had mild NPDR, 108 (6.6%) had moderate NPDR, 11 (0.6%) had severe NPDR, and 172 (10.4%) had proliferative DR. Among those patients with DR, 44 (2.7%) presented macular edema. The clinical and demographic characteristics of the studied population are shown in Table 1.

**Table 1:** Demographic and clinical data of the study population

Variable	
N	1644
Demographic characteristics	
Gender, female, n (%)	917 (55.8)
Age, years	30.1 ± 12.0
Age of diagnosis, years	14.6 ± 8.9
Duration of diabetes, years	15.3 ± 9.3
Years of formal education, years	12.3 ± 3.8
Economic status, n (%)	
High	49 (3.0)
Medium	745 (45.3)
Low	795 (48.4)
Very low	55 (3.3)
Clinical data	
HbA1c (%)	9.0 ± 2.1
HbA1c, mean (SD), mmol/mol	74.5 ± 23.1
Diet adherence > 80%, n (%)	846 (58.4)
Insulin regimens, n (%)	
Intermediate or long acting	80 (4.9)
Intermediate/long plus short acting	1.510 (91.8)
CSII	54 (3.3)
Serum uric acid, mean (SD), mg/dL	5.1 ± 1.8
HDL cholesterol, mean (SD), mg/dL	56.8 ± 19.1
LDL cholesterol, mean (SD), mg/dL	109.8 ± 40.8
Triglycerides, mean (SD), mg/dL	109.8 ± 84.6
BMI, mean (SD), kg/m^2^	24.2 ± 4.2
Arterial hypertension, yes, n (%)	288 (17.5)
Macrovascular disease, yes n (%)	57 (3.5)
Use of an angiotensin-converting enzyme (ACE) inhibitor, yes, n (%)	437 (26.6)
Current smoker, yes, n (%)	86 (5.6)
Chronic kidney disease, yes, n (%)	289 (16.4)
Diabetic retinopathy, n (%)	
Absent	1055 (64.2)
Mild non-proliferative	298 (18.1)
Moderate non-proliferative	108 (6.6)
Severe non-proliferative	11 (0.6)
Proliferative	172 (10.4)
Macular edema, yes, n (%)^†^	44 (2.7)

Notes: Data are presented as numbers (percentages) or means ± SD (standard deviation)

^†^Of the total patiens with DR (mild, moderate, severe and proliferative), 44 presented macular edema

*Abbreviations***:** *BMI* body mass index, *HbA1c* glycated hemoglobin, *CSII* continuous subcutaneous insulin infusion, *LDL-c* low density lipoprotein cholesterol, *HDL* high density lipoprotein cholesterol

**Fig. 1:**
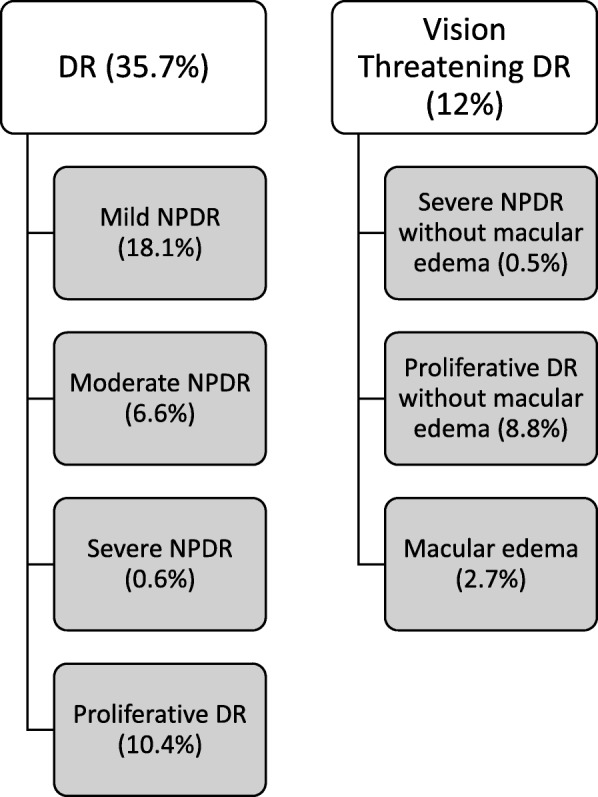
Distribution of patients according to the categories of Diabetic retinopathy DR: diabetic retinopathy; NPDR: non-proliferative diabetic retinopathy

The distribution of patients in the sub-group of VTDR was as follows: 198 (12%) VTDR and 1446 (88%) non-VTDR (Fig. 1).

### Social-demographic, clinical and laboratory risk factors for DR

The descriptive analysis has shown that patients with DR, were older, had longer diabetes duration, less years of formal education, higher BMI, SUA, HbA1c and triglycerides, were more likely to be users of angiotensin-converting enzyme (ACE) inhibitors, and had a higher prevalence of hypertension, macrovascular disease and CKD (*p* < 0.001 for all comparisons) than patients without DR. Levels of serum uric acid were higher in men than in women (mean 5.57 vs. 4.69 mg/dl; *p* < 0.001). Retinopathy was also not associated with gender, economic status, smoking status, HDL or LDL cholesterol levels (Table 2).

**Table 2:** Demographic, clinical and laboratory data stratified by diabetic retinopathy type

Variables	Present	Absent	*p*-Value	VTDR	Non VTDR	*p*-Value
N, (%)	589 (35.8)	1055 (64.2)		198 (12.1)	1446 (87.9)	
Demographic data						
Gender, female, n (%)	343 (58.2)	574 (54.4)	0.1	114 (57.6)	803 (55.5)	0.5
Age, mean (SD), years	35.77 ± 11.56	26.86 ± 11.11	< 0.001	37.35 ± 11.07	29.05 ± 11.84	< 0.001
Duration of diabetes, mean (SD), years	20.26 ± 9.18	12.62 ± 8.15	< 0.001	23.28 ± 9.02	14.25 ± 8.77	< 0.001
Years of formal education, mean (SD), years	11.92 ± 4.22	12.43 ± 3.55	0.01	12.05 ± 4.22	12.27 ± 3.75	0.4
Economic status, n (%)						
High	14 (2.4)	35 (3.3)	0.3	5 (2.5)	44 (3.0)	0.1
Medium	261 (44.3)	484 (45.9)		86 (43.4)	659 (45.6)	
Low	289 (49.1)	506 (48.0)		95 (48.0)	700 (48.4)	
Very low	25 (4.2)	30 (2.8)		12 (6.1)	43 (3.0)	
Clinical data						
HbA1c mg/dl (%)	9.27 (2.22)	8.86 (2.04)	< 0.001	9.10 (2.06)	8.99 (2.12)	0.4
HbA1c, mean (SD), mmol/mol	77.84 ± 24.27	73.34 ± 22.33	< 0.001	76.03 ± 22.53	74.8 ± 23.22	0.4
Serum uric acid, mean (SD), mg/dL	5.58 ± 2.09	4.81 ± 1.63	< 0.001	6.09 ± 2.04	4.95 ± 1.77	< 0.001
Hypertension, yes, n (%)	197 (33.4)	91 (8.6)	< 0.001	85 (42.9)	203 (14.1)	< 0.001
Triglycerides, mean (SD), mg/dL	102.99 ± 76.59	121.98 ± 96.30	< 0.001	122.46 ± 88.96	108.07	0.02
HDL cholesterol, mean (SD), mg/dL	57.84 ± 19.89	56.21 ± 18.63	0.1	54.50 ± 17.53	57.11 ± 19.29	0.56
LDL cholesterol, mean (SD), md/dL	111.90 ± 43.91	108.31 ± 38.98	0.09	113.37 ± 38.46	110.56 ± 41.28	0.3
BMI, mean (SD), kg/m2	25.09 ± 4.69	23.65 ± 3.81	< 0.001	25.00 ± 4.90	24.05 ± 4.08	0.01
Macrovascular disease, yes n (%)	39 (6.6)	18 (1.7)	< 0.001	13 (6.6)	44 (3.1)	0.01
Use of an angiotensin converting enzyme (ACE) inhibitor, yes, n (%)	269 (45.7)	168 (16.0)	< 0.001	112 (22.6)	325 (56.6)	< 0.001
Current smoker, yes, n (%)	38 (6.5)	48 (4.5)	0.09	14 (7.1)	72 (5.0)	0.2
Chronic kidney disease, yes, n (%)	159 (27.4)	105 (10.1)	< 0.001	83 (42.8)	181 (12.7)	< 0.001

The data are presented as numbers (percentages) or means ± SD (standard deviation). The *p* value compares differences between the groups using Student’s t-test. *BMI* body mass index, *HbA1c* glycated hemoglobin, *LDL-c* low density lipoprotein cholesterol, high density lipoprotein cholesterol, *VTDR* vision-threatening diabetic retinopathy

Descriptive analysis of clinical and laboratory features of individuals, stratified by traditional classification of DR (non proliferative mild, moderate, severe and proliferative), are shown as an Additional file 1: Table S1.

The final adjusted model of multivariate binomial logistic regression is described in Table 3. The independent variables included in the model explained 32.5% (Nagelkerke *R*^*2*^) of the variance for the presence of any type of DR. The highest odds were 2.26 for macrovascular disease (CI95% 1.08–4.71; *p =* 0.03) and 1.68 for arterial hypertension (CI95% 1.13–2.5; *p* = 0.01). Age, duration of DM, HbA1c and serum uric acid were also significant predisposing factors to DR.

**Table 3:** Final adjusted logistic regression model

	b	OR	CI 95%		*p* value
Present vs. Absent					
Age, years	0.028	1.03	1.02	1.04	< 0.001
Duration of DM, years	0.067	1.07	1.05	1.09	< 0.001
HbA1c (%)	0.216	1.24	1.17	1.32	< 0.001
Serum uric acid, mg/dL	0.196	1.22	1.13	1.31	< 0.001
Use of an angiotensin-converting enzyme (ACE) inhibitor, yes	0.563	1.78	1.26	2.43	< 0.001
Macrovascular disease, yes	0.813	2.26	1.08	4.71	0.03
Arterial hypertension, yes	0.521	1.68	1.13	2.5	0.01
VTDR vs. Non-VTDR					
Duration of DM, years	0.075	1.08	1.06	1.1	< 0.001
HbA1c %	0.105	1.11	1.02	1.21	0.014
Serum uric acid, mg/dL	0.219	1.25	1.13	1.37	< 0.001
Use of an angiotensin-converting enzyme (ACE) inhibitor yes	0.774	2.17	1.51	3.11	< 0.001
LDL cholesterol, mg/dL	−0.01	0.99	0.99	1	0.019
Chronic kidney disease, yes	0.925	2.52	1.69	3.77	< 0.001

*VTDR* vision-threatening diabetic retinopathy, *HbA1c* glycated hemoglobin, *LDL-c* low density lipoprotein cholesterol, *b* coefficient for logistic regression; *OR* odds ratio, *CI 95%* 95% confidence interval

The crude odds ratio (OR) at univariate level can be accessed as Additional file 2: Table S2 and Additional file 3: Table S3.

### Social-demographic, clinical and laboratory risk factors for VTDR DR (severe NPDR, proliferative DR and macular edema)

Descriptive analysis showed that patients with VTDR were older (*p* < 0.001), with a longer diabetes duration (*p* < 0.001), had higher levels of SUA (*p* < 0.001), triglycerides (*p* = 0,02) and BMI (*p* = 0.01), were more likely to be users of an ACE inhibitor (*p* < 0.001), to have hypertension (*p* < 0.001), macrovascular disease (*p* < 0.001) and CKD (*p* < 0.001), than patients without VTDR. We found no association with gender, number of years of formal education, economic status, levels of HbA1c, HDL or LDL cholesterol, or smoking status (Table 2).

The independent variables included in the final adjusted multivariate binomial logistic regression model explained 27.3% (Nagelkerke *R*^*2*^) of the variance for the presence of VTDR. The highest odds were 2.52 for CKD (CI95% 1.69–3.77; *p <* 0.001) and 2.17 for use of an ACE inhibitor (CI95% 1.51–3.11; *p* < 0.001). Duration of DM, HbA1c and serum uric acid were also significant predisposing factors to VTDR. High levels of LDL cholesterol is related to low risk of VTDR. Data are shown in Table 3.

## Discussion

This was the first multicenter study in Brazil to estimate the prevalence of DR in patients with T1D. We also analyzed risk factors for the development of any DR or VTDR in this population. The prevalence of any DR was 35%, of which 12% presented VTDR that required immediate referral for treatment evaluation.

This result is consistent with the worldwide estimates of the overall prevalence of DR (34.6%) but we found a slightly higher prevalence of VTDR (12% vs. 10%) [1, 2, 19]. However, it is important to emphasize that the prevalence of DR varies with geography and ethnicity worldwide [4]. Socioeconomic and genetic factors may influence this variability. In Brazil, we found only one study that described the prevalence of diabetic retinopathy in patients with T1D [20]. However, this was a small study conducted in a selected population limited to a single hospital and showed a higher DR prevalence than in our sample (40.4% vs 35.7%, respectively).

Although in our sample the majority of patients were using intensive insulin therapeutic regimens with less than 5% using continuous insulin infusion, we found high levels of HbA1c, which could be explained by the lack of adherence to insulin therapeutic regimens [12] as well as low adherence to diet [21]. However, other factors such as socioeconomic status and educational level could be related to the observed higher levels of HbA1c as recently demonstrated [22, 23].

In our study, three variables were frequently associated with retinopathy regardless of DR group: longer duration of diabetes, higher levels of HbA1c and SUA. Arterial hypertension was associated with DR in patients with any kind of DR. Multiple studies have consistently shown that a longer duration of diabetes, poor glycemic control and hypertension are positively associated with the development of DR [11, 24]. The finding of a significant association between HbA1c and DR is consistent with the results found on DCCT [7] and the Wisconsin epidemiologic study of DR (WESDR) [25]. The use of ACE inhibitors was also associated with DR in both groups. We believe that the use of ACE inhibitors was found to be significant because of its frequent use regarding the treatment of arterial hypertension.

Another interesting finding was the association of DR, independent of its severity, with SUA levels. This result is consistent with observations from a number of studies suggesting an association between SUA levels and vascular complications in patients with T1D, including DR [26, 27]. Some cross-sectional and prospective studies, conducted in Thailand and Japan [28, 29] have also shown association between uric acid serum levels and DR in patients with type 2 diabetes. However, in Japan this association was only observed in men [29]. Although our study found higher levels of SUA in men, gender was not a significant variable for DR or VTDR. Possible mechanisms that may explain this association include the pro-inflammatory and pro-oxidant effects of SUA, which can lead to endothelial damage. Uric acid, a product of purine metabolism, usually acts as an antioxidant. However, paradoxically, it can also act as a pro-oxidant because reactive oxygen species are generated during its production [30, 31]. A previous experimental study suggests that higher levels of SUA may stimulate the production of inflammatory cytokines [32].

In this current study, the presence of VTDR showed a significant association with CKD. A number of studies provide evidence that the presence of DR may indicate patients at risk of diabetic nephropathy [33, 34]. Retinopathy and nephropathy are microvascular complications that may reflect similar vascular changes with common mechanisms. Hyperglycemia activates intracellular signaling pathways, leading to oxidative stress, endothelial injury and the overproduction of inflammatory markers [35]. Increased vascular permeability, thickening of the basement membrane and muscular layers are common in both retinopathy and nephropathy [33].

As in other studies, our results have shown a significant association between any type of DR and macrovascular disease. This association may be related to the fact that both conditions share similar risk factors, such as poor glycemic control and hypertension [36, 37].

Patients with higher levels of LDL-cholesterol showed lower risk of VTDR. We believe that patients with a high level of LDL-cholesterol use more frequently statins or fibrates and these drugs could have a protector effect regarding DR. The impact of statins and fibrates on reducing DR is still a matter of debate. However, studies on patients with type 2 diabetes have shown, that the use of fibrates and statins can prevent the progression of DR and reduce the requirements of laser therapy, independent of the effect on cholesterol control or levels [38, 39].

The strengths of our study are as follows: first, to the best of our knowledge, this present large and multicenter study with a multi-ethnic population is the first to determine the prevalence of DR in patients with T1D in Brazil. Second, the fact that T1D patients are younger and have a less comorbidities than those with type 2 diabetes, allows a more reliable assessment of the risk factors for DR. Third, we categorized DR as VTDR, consistent with the epidemiological literature, thus allowing an analysis of patients at greater risk of visual impairment.

Our study has also some limitations. First, this was a cross-sectional study; hence, it is not possible to establish a true cause and effect relationship based on the data. Second, we used the self-reported diagnosis of arterial hypertension; thus, the prevalence of this disease may have been underestimated. However, we consider that this type of self-reported information exhibits substantial agreement between the questionnaire responses and medical records regarding information for hypertension (approximately 82% sensitivity and 92.2% specificity) [40]. Third, T1D was diagnosed according to the clinical definition assigned by a physician without evaluation of islet cell antibodies and c-peptide as well as genetic evaluation for MODY. However, clinical definition of T1D is used in most epidemiological studies. Fourth, although the gold standard methodology for diagnosis of DR is the 7-field stereoscopic photographs established by ETDRS [41], both BIO and retinography are valid strategies for the screening of DR. Previous study from our group has shown significant agreement between the two methods for DR classification [9]. Due to logistic issues, assessment of inter observer variability on screening of DR was not conducted. Fifth, we have included patients with less than 5 years of DM, of whom eight had DR, and seven of these with mild NPDR. Although ADA consensus recommends screening for DR in patients with more than 5 years of T1D, some studies, as DCCT, revealed that patients with T1D and less than 5 years of disease could present DR [42].

## Conclusion

In summary, our data have shown a high prevalence of DR among Brazilian T1D patients consistent with the worldwide prevalence of this condition. We found a high proportion of patients with VTDR. This highlights the need for improving access to eye care and DR screening programs in Brazil, which would be beneficial for the prevention of visual impairment and loss. In addition, our data have shown that besides the traditional risk factors (duration of diabetes, poor glycemic control and hypertension) SUA levels has emerged as a potential marker of DR. This emphasizes the need to continuously seek the best clinical control for patients with T1D to prevent this disabling complication. Furthermore, future studies addressing the relationship between DR and SUA levels are needed.

## Additional files

Additional file 1: **Table S1.** Demographic, clinical and laboratory data stratified by diabetic retinopathy type. (DOCX 20 kb)

Additional file 2: **Table S2.** Multivariate analysis of diabetic retinopathy (Present vs. Absent). (DOCX 21 kb)

Additional file 3: **Table S3.** Multivariate analysis of diabetic retinopathy (Vision threatening vs. no vision threatening). (DOCX 102 kb)

## Abbreviations

ACE: Angiotensin-converting enzyme
ADA: American diabetes association
BIO: Binocular indirect ophthalmoscopy
BMI: Body mass index
CKD: Chronic kidney disease
CSII: Continuous subcutaneous insulin infusion
DCCT: Diabetes Control and Complications Trial
DR: Diabetic retinopathy
GFR: Glomerular filtration rate
HbA1c: Glycated hemoglobin
HDL-c: High density lipoprotein cholesterol
LDL-c: Low density lipoprotein cholesterol
NPDR: Non-proliferative diabetic retinopathy
PDR: Proliferative diabetic retinopathy
SUA: Serum uric acid
T1D: Type 1 diabetes
TG: Triglycerides
VTDR: Vision threatening diabetic retinopathy
