# Supplementary material for: Current epidemiology of diabetic retinopathy in patients with type 1 diabetes: a national multicenter study in Brazil
Source: BMC Public Health. 2018 Aug 8;18:989. doi: 10.1186/s12889-018-5859-x (PMC6083618; doi:10.1186/s12889-018-5859-x)
Supplement: Supplementary file 1 — Table S1. Demographic, clinical and laboratory data stratified by diabetic retinopathy type. (DOCX 20 kb) [file 12889_2018_5859_MOESM1_ESM.docx]

| Table S1. Demographic, clinical and laboratory data stratified by diabetic retinopathy type | | | | | | |
| --- | --- | --- | --- | --- | --- | --- |
| **Variables** | **Absent** | **Mild NPDR** | **Moderate NPDR** | **Severe NPDR** | **PDR** | **P-Value** |
| N (%) | 1055 (64.2) | 298 (18.1) | 108 (6.6) | 11 (0.7) | 172 (10.4) |  |
| ***Demographic data*** |  |  |  |  |  |  |
| Gender, female n(%) | 574 (54.4) | 176 (59.1) | 62 (57.4) | 9 (81.8) | 96 (55.8) | 0.2 |
| Age, mean (SD), years | 26.86 ± 11.11 | 34.33 ± 11.84 | 36.11 ± 11.23 | 28.64 ± 8.22 | 38.5 ± 10.89 | <0.001 |
| Duration of diabetes, mean (SD), years | 12.62 ± 8.15 | 18.13 ± 8.79 | 19.96 ± 8.60 | 19.73 ± 8.23 | 24.05 ± 9.10 | <0.001 |
| Years of formal education, mean (SD), years | 12.43 ± 3.55 | 12.12 ± 4.26 | 11.27 ± 4.14 | 12.27 ± 3.03 | 11.95 ± 4.26 | 0.02 |
| Economic status, n (%) |  |  |  |  |  | 0.09 |
| High | 35 (3.3) | 5 (1.7) | 4 (3.7) | 0 | 5 (2.9) |  |
| Medium | 484 (45.9) | 139 (46.6) | 41 (38.0) | 4 (36.4) | 77 (44.8) |  |
| Low | 506 (48.0) | 147 (49.3) | 56 (51.9) | 5 (45.5) | 81 (47.1) |  |
| Very low | 30 (2.8) | 7 (2.3) | 7 (6.5) | 2 (18.2) | 9 (5.2) |  |
| ***Clinical data*** |  |  |  |  |  |  |
| HbA1c mg/dL, % | 8.86 ± 2.04 | 9.21 ± 2.17 | 9.97 ± 2.70 | 9.5 ± 2.52 | 8.91 ± 1.81 | <0,001 |
| HbA1c, mean (SD), mmol/mol | 73.34 ± 22.33 | 77.23 ± 23.82 | 85.47 ± 29.59 | 80.42 ± 27.64 | 73.9 ± 19.80 | <0.001 |
| Serum uric acid, mean (SD), mg/dL | 4.81 ± 1.63 | 5.24 ± 1.99 | 5.62 ± 2.15 | 6.27 ± 1.92 | 6.1 ± 2.11 | <0.001 |
| Hypertension, n(%) | 91 (8.6) | 75 (25.2) | 41 (38.0) | 3 (27.3) | 78 (45.3) | <0.001 |
| Triglycerides, mean (SD), mg/dL | 102.99 ± 76.59 | 121.52 ± 106.01 | 127.36 ± 82.00 | 158.1 ± 148.13 | 117.3 ± 82.49 | <0.001 |
| HDL cholesterol, mean (SD), mg/dL | 56.21 ± 18.63 | 59.54 ± 20.56 | 59.28 ± 20.97 | 57.07 ± 20.10 | 54.02 ± 17.50 | 0.01 |
| LDL cholesterol, mean (SD), mg/dL | 110.2 ± 42.26 | 112.96 ± 36.77 | 110.67 ± 41.90 | 125.18 ± 79.78 | 110.91 ± 33.92 | 0.6 |
| BMI, mean (SD), kg/m^2^ | 23.65 ± 38.81 | 24.99 ± 4.55 | 25.41 ± 4.44 | 23.1 ± 4.53 | 25.19 ± 5.06 | <0.001 |
| Macrovascular disease, yes, n (%) | 18 (1.7) | 15 (5.0) | 11 (10.2) | 1 (9.1) | 12 (7.0) | <0.001 |
| Use of an angiotensin-converting enzyme (ACE) inhibitor, yes, n(%) | 168 (16.0) | 108 (36.4) | 54 (50.0) | 9 (81.8) | 98 (57.0) | <0.001 |
| Current smoker, yes, n(%) | 48 (4.5) | 15 (5.1) | 10 (9.3) | 1 (9.1) | 12 (7.0) | 0.2 |
| Chronic kidney disease, yes, n (%) | 105 (10.1) | 54 (18.2) | 27 (25.7) | 4 (40.0) | 74 (43.8) | <0.001 |
| The data are presented as numbers (percentages), means ± SD (standard deviation). The p value compares differences between the groups using one-way ANOVA. BMI: body mass index; HbA1c: glycated hemoglobin; LDL-c: low density lipoprotein cholesterol; HDL: high density lipoprotein cholesterol; NPDR = Non-proliferative diabetic retinopathy. PDR = Proliferative diabetic retinopathy | | | | | | |
