# Supplementary material for: Current epidemiology of diabetic retinopathy in patients with type 1 diabetes: a national multicenter study in Brazil
Source: BMC Public Health. 2018 Aug 8;18:989. doi: 10.1186/s12889-018-5859-x (PMC6083618; doi:10.1186/s12889-018-5859-x)
Supplement: Supplementary file 2 — Table S2. Multivariate analysis of diabetic retinopathy (Present vs. Absent). (DOCX 21 kb) [file 12889_2018_5859_MOESM2_ESM.docx]

| Table S2: Multivariate analysis of diabetic retinopathy (Present *vs.* Absent) | | | | | | | | |
| --- | --- | --- | --- | --- | --- | --- | --- | --- |
|  | **Unadjusted** | **Model1** | **Model2** | **Model3** | **Model4** | **Model5** | **Model6** | **Model7** |
| Duration of DM, years | 1.070 (1.051-1.089) | 1.101 (1.086-1.117) | 1.084 (1.069-1.100) | 1.096 (1.080-1.113) | 1.095 (1.079-1.112) | 1.070 (1.052-1.089) | 1.070 (1.051-1.089) | 1.069 (1.051-1.088) |
| Use of an angiotensin-converting enzyme (ACE) inhibitor | 1.782 (1.280-2.480) |  | 2.983 (2.306-3.858) | 2.857 (2.198-3.714) | 2.620 (2.007-3.419) | 2.248 (1.708-2.957) | 1.793 (1.295-2.482) | 1.755 (1.266-2.433) |
| HbA1c (%) | 1.227 (1.152-1.308) |  |  | 1.213 (1.144-1.285) | 1.212 (1.143-1.285) | 1.238 (1.165-1.314) | 1.240 (1.167 -1.316) | 1.241 (1.168-1.317) |
| Serum uric acid, mg/dL | 1.210 (1.108-1.321) |  |  |  | 1.213 (1.131-1.301) | 1.228 (1.144-1.318) | 1.218 (1.134-1.309) | 1.217 (1.132-1.307) |
| Age, years | 1.025 (1.011-1.039) |  |  |  |  | 1.033 (1.020-1.46) | 1.029 (1.016-1.043) | 1.028 (1.015-1.042) |
| Arterial hypertension, yes | 1.526 (1.013-2.298) |  |  |  |  |  | 1.678 (1.131-2.488) | 1.684 (1.134-2.502) |
| Macrovascular disease, yes | 2.231 (1.061-4.692) |  |  |  |  |  |  | 2.256 (1.081-4.709 |
| Gender | 0.860 (0.655-1.128) |  |  |  |  |  |  |  |
| Years of formal education | 0.990 (0.956-1.026) |  |  |  |  |  |  |  |
| BMI, kg/m^2^ | 1.013 (0.981-1.046) |  |  |  |  |  |  |  |
| Current smoker | 1.231 (0.722-2.100) |  |  |  |  |  |  |  |
| LDL cholesterol, mean (SD), mg/dL | 0.999 (0.996-1.002) |  |  |  |  |  |  |  |
| HDL cholesterol, mg/dL | 1.004 (0.997-1.010) |  |  |  |  |  |  |  |
| Triglycerides, mg/dL | 1.001 (0.998-1.003) |  |  |  |  |  |  |  |
| Chronic kidney disease | 1.262 (0.873-1.824) |  |  |  |  |  |  |  |
| Economic status, n (%) |  |  |  |  |  |  |  |  |
| High | 0.878 (0.316-2.441) |  |  |  |  |  |  |  |
| Medium | 1.056 (0.522-2.137) |  |  |  |  |  |  |  |
| Low | 1.106 (0.559-2.189) |  |  |  |  |  |  |  |
| Data are presented as: odds ratio and CI 95% ( 95% confidence interval). Abbreviations: DM, diabetes Mellitus; ACE, angiotensin-converting enzyme; BMI, body mass index; HbA1c, glycated hemoglobin; LDL-c, low density lipoprotein cholesterol; HDL, high density lipoprotein cholesterol.  Unadjusted: non-adjusted (forward:wald model)  Model 1: after adjustment for duration of DM, use of ACE inhibitor, HbA1c, serum uric acid, age, arterial hypertension, macrovascuar disease , chronic kidney disease,LDL, gender, years of formal education, BMI, current smoker, HDL, triglycerides, and economic status, did not persisted in the model.  Model 2: after adjustment for duration of DM + use of ACE inhibitor, HbA1c, serum uric acid, age, arterial hypertension, macrovascuar disease , chronic kidney disease,LDL, gender, years of formal education, BMI, current smoker, HDL, triglycerides, and economic status, did not persisted in the model.  Model 3: after adjustment for duration of DM + use of ACE inhibitor + HbA1c, serum uric acid, age, arterial hypertension, macrovascuar disease , chronic kidney disease,LDL, gender, years of formal education, BMI, current smoker, HDL, triglycerides, and economic status, did not persisted in the model.  Model 4: after adjustment for duration of DM + use of ACE inhibitor + HbA1c + serum uric acid, age, arterial hypertension, macrovascuar disease , chronic kidney disease,LDL, gender, years of formal education, BMI, current smoker, HDL, triglycerides, and economic status, did not persisted in the model.  Model 5: after adjustment for duration of DM + use of ACE inhibitor + HbA1c + serum uric acid + age, arterial hypertension, macrovascuar disease , chronic kidney disease,LDL, gender, years of formal education, BMI, current smoker, HDL, triglycerides, and economic status, did not persisted in the model.  Model 6: after adjustment for duration of DM + use of ACE inhibitor + HbA1c + serum uric acid + age + arterial hypertension, macrovascuar disease , chronic kidney disease,LDL, gender, years of formal education, BMI, current smoker, HDL, triglycerides, and economic status, did not persisted in the model.  Model 7: after adjustment for duration of DM + use of ACE inhibitor + HbA1c + serum uric acid + age + arterial hypertension + macrovascuar disease , chronic kidney disease,LDL, gender, years of formal education, BMI, current smoker, HDL, triglycerides, and economic status, did not persisted in the model. | | | | | | | | |
