# Supplementary material for: Current epidemiology of diabetic retinopathy in patients with type 1 diabetes: a national multicenter study in Brazil
Source: BMC Public Health. 2018 Aug 8;18:989. doi: 10.1186/s12889-018-5859-x (PMC6083618; doi:10.1186/s12889-018-5859-x)
Supplement: Supplementary file 3 — Table S3. Multivariate analysis of diabetic retinopathy (Vision threatening vs. no vision threatening). (DOCX 102 kb) [file 12889_2018_5859_MOESM3_ESM.docx]

| Table S3: Multivariate analysis of diabetic retinopathy (Vision threatening *vs.* no vision threatening) | | | | | | | |
| --- | --- | --- | --- | --- | --- | --- | --- |
|  | **Unadjusted** | **Model1** | **Model2** | **Model3** | **Model4** | **Model5** | **Model6** |
| Duration of DM, years | 1.084 (1.058-1.111) | 1.095 (1.078-1.113) | 1.093 (1.075-1.112) | 1.080 (1.061-1.099) | 1.073 (1.054-1.092) | 1,078 (1,058-1,098) | 1,078 (1,058-1,098) |
| Serum uric acid, mg/dL | 1.304 (1.164-1.460) |  | 1.351 (1.241-1.470) | 1.314 (1.205-1.433) | 1.223 (1.115-1.341) | 1.222 (1.115-1.340) | 1.245 (1.113-1.367) |
| Use of an angiotensin-converting enzyme (ACE) inhibitor | 1.883 (1.216-2.918) |  |  | 2.451 (1.726-3.481) | 2.317 (1.624-3.306) | 2.271 (1.591-3.242) | 2.169 (1.513-3.108) |
| Chronic kidney disease | 2.314 (1.499-3.572) |  |  |  | 2.361 (1.591-3.505) | 2.353 (1.585-3.493) | 2.521 (1.687-3.767) |
| HbA1c (%) | 1.121 (1.026-1.225) |  |  |  |  | 1.090 (1.004-1.184) | 1.111 (1.022-1.207) |
| LDL cholesterol, mg/dL | 0.995 (0.990-1.000) |  |  |  |  |  | 0.994 (0.990-0.999) |
| Gender | 0.849 (0.576-1.249) |  |  |  |  |  |  |
| Age, years | 0.996 (0.976-1.016) |  |  |  |  |  |  |
| Years of formal education | 1.004 (0.957-1.053) |  |  |  |  |  |  |
| BMI, kg/m^2^ | 0.979 (0.938-1.022) |  |  |  |  |  |  |
| Current smoker | 1.902 (0.970-3.728) |  |  |  |  |  |  |
| HDL cholesterol, mg/dL | 0.991 (0.981-1.001) |  |  |  |  |  |  |
| Triglycerides, mg/dL | 0.998 (0.994-1.001) |  |  |  |  |  |  |
| Arterial hypertension, yes | 1.460 (0.898-2.374) |  |  |  |  |  |  |
| Macrovascular disease, yes | 0.694 (0.305-1.577) |  |  |  |  |  |  |
| Economic status, n (%) |  |  |  |  |  |  |  |
| High | 0.719 (0.179-2.891) |  |  |  |  |  |  |
| Medium | 0.652 (0.267-1.589) |  |  |  |  |  |  |
| Low | 0.706 (0.300-1.661) |  |  |  |  |  |  |
| Data are presented as: odds ratio and CI 95% ( 95% confidence interval). Abbreviations: DM, diabetes Mellitus; ACE, angiotensin-converting enzyme; BMI, body mass index; HbA1c, glycated hemoglobin; LDL-c, low density lipoprotein cholesterol; HDL, high density lipoprotein cholesterol.  Unadjusted: non-adjusted (forward:wald model)  Model 1: after adjustment for duration of DM, serum uric acid, use of ACE inhibitor, chronic kidney disease, HbA1c,LDL, gender, age, years of formal education, BMI, current smoker, HDL, triglycerides, arterial hypertension, macrovascuar disease and economic status, did not persisted in the model.  Model 2: after adjustment for duration of DM + serum uric acid, use of ACE inhibitor, chronic kidney disease, HbA1c, LDL, gender, age, years of formal education, BMI, current smoker, HDL, triglycerides, arterial hypertension, macrovascuar disease and economic status, did not persisted in the model.  Model 3: after adjustment for duration of DM + serum uric acid + use of ACE inhibitor, chronic kidney disease, HbA1c, LDL, gender, age, years of formal education, BMI, current smoker, HDL, triglycerides, arterial hypertension, macrovascuar disease and economic status, did not persisted in the model.  Model 4: after adjustment for duration of DM +serum uric acid + use of ACE inhibitor + chronic kidney disease, HbA1c, LDL, gender, age, years of formal education, BMI, current smoker, HDL, triglycerides, arterial hypertension, macrovascuar disease and economic status, did not persisted in the model.  Model 5: after adjustment for duration of DM + serum uric acid + use of ACE inhibitor + chronic kidney disease + HbA1c, LDL, gender, age, years of formal education, BMI, current smoker, HDL, triglycerides, arterial hypertension, macrovascuar disease and economic status, did not persisted in the model.  Model 6: after adjustment for duration of DM + serum uric acid + use of ACE inhibitor + chronic kidney disease + HbA1c + LDL, gender, age, years of formal education, BMI, current smoker, HDL, triglycerides, arterial hypertension, macrovascuar disease and economic status, did not persisted in the model. | | | | | | | |
|  |  |  |  |  |  |  |  |
